# Supplementary material for: Non-autonomy of age-related morphological changes in the C. elegans germline stem cell niche
Source: bioRxiv. 2025 Jun 16:2025.06.13.658151. Preprint. [Version 1] doi: 10.1101/2025.06.13.658151 (PMC12262334; doi:10.1101/2025.06.13.658151)
Supplement: Supplementary file 1 [file media-1.pdf]

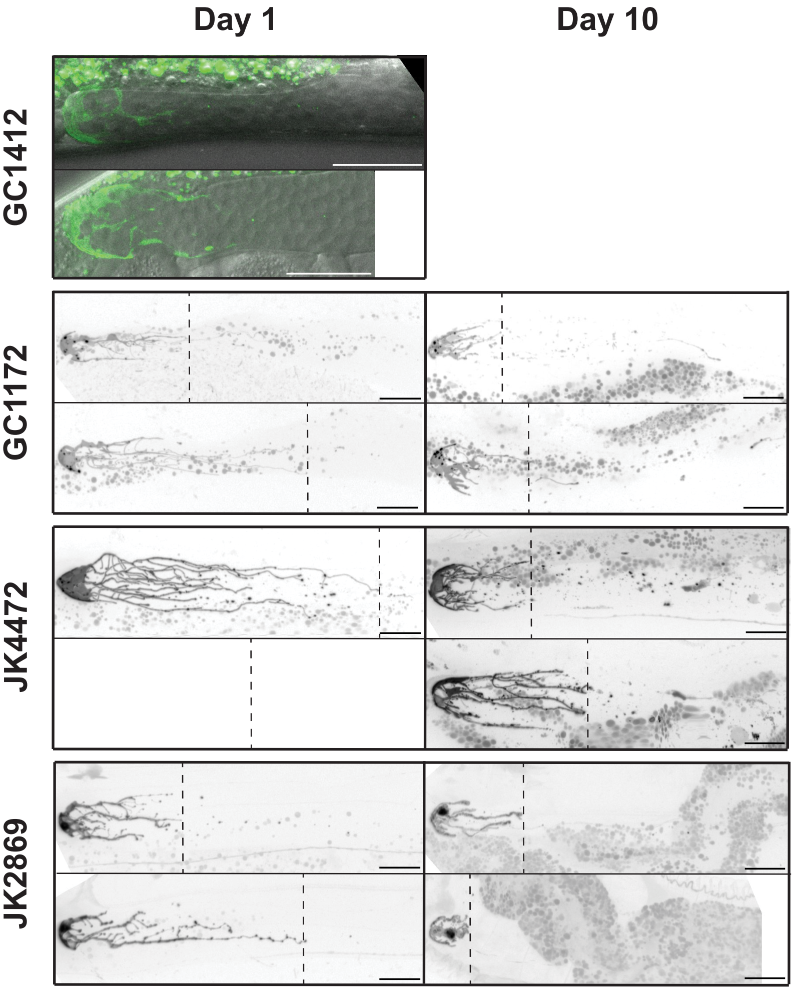

Strain	Relevant Genotype	Relevant Features
GC1412	***naSi8*** Single copy insertion of *lag-2p::GFP-PH*	Dim expression; nucleus not visible
GC1172	***naIs37*** Low-copy insertion of *lag-2p::PH-mCherry*	*mCherry* aggregates; dim expression; nucleus not visible
JK4472	***qIs154*** Insertion of *lag-2p::Myr-tdTomato*	Strong localization to the plasma membrane; variable intensity; high detail; nucleus not visible
JK2869	***qIs57*** Insertion of *lag-2p::GFP*	Strong fluorescence signal throughout the cell; high and consistent intensity; moderate detail; clearly visible nucleus

Figure S1

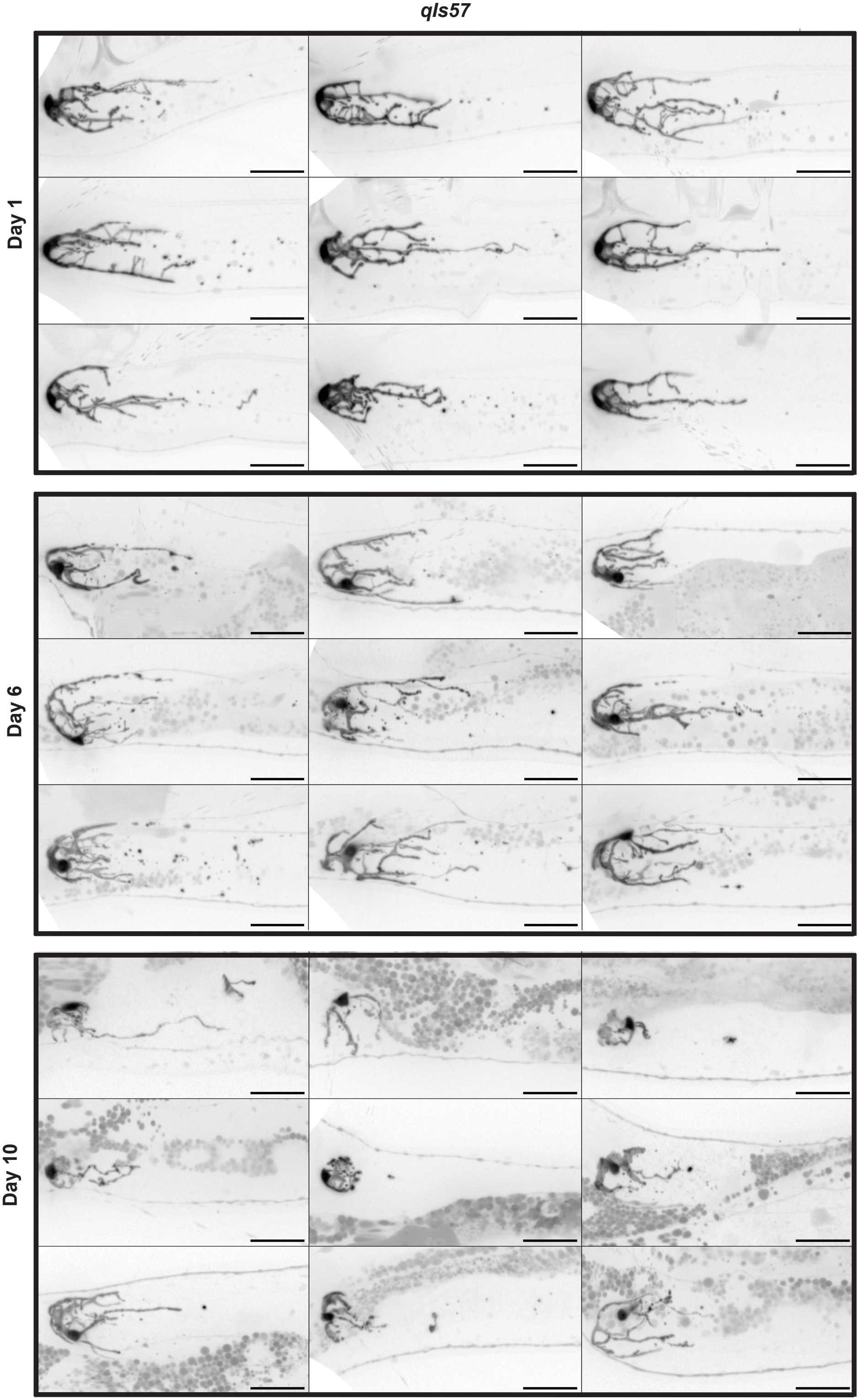

Figure S2

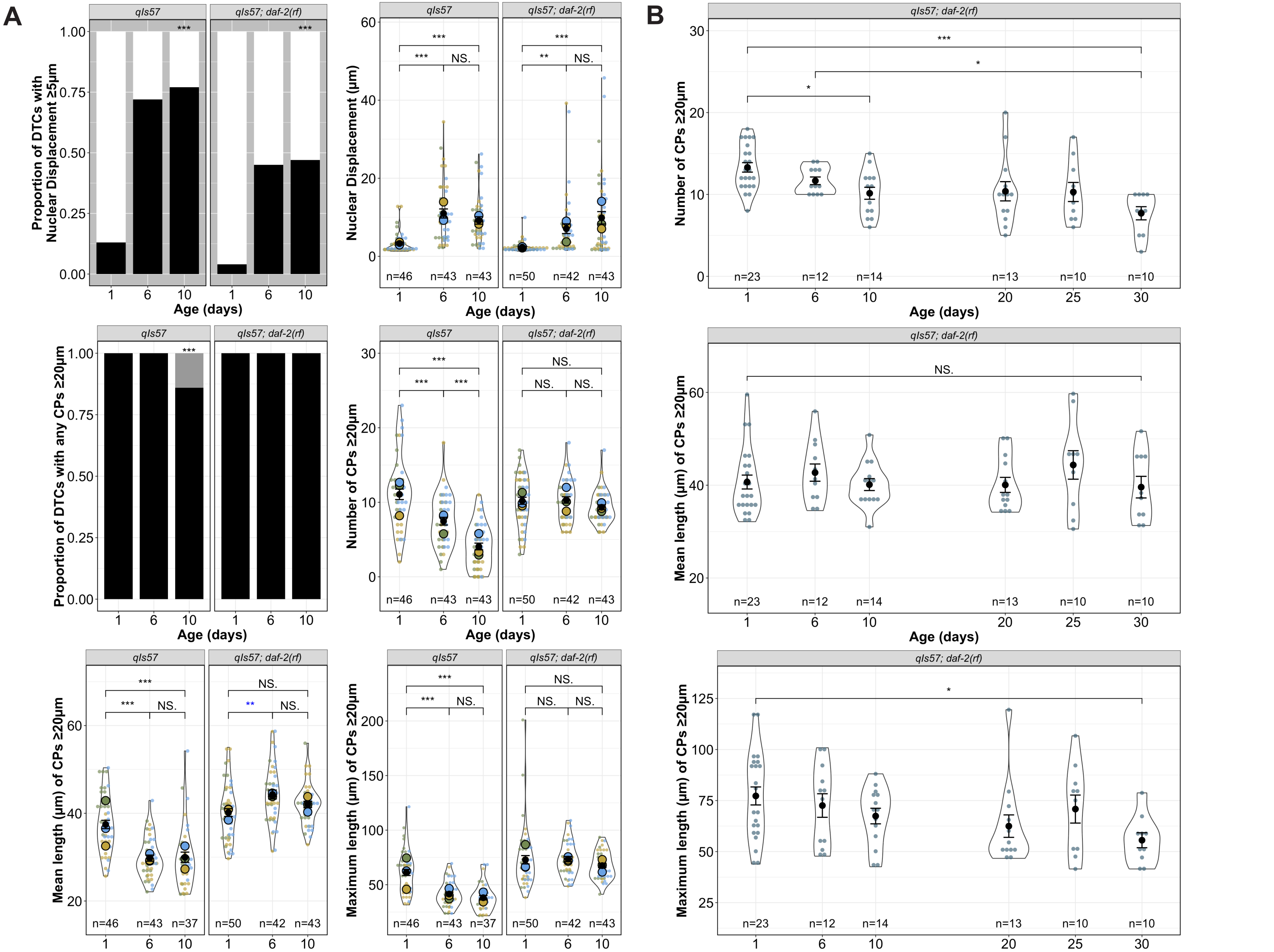
A
B
qIs57
qIs57; daf-2(rf)
Proportion of DTCs with Nuclear Displacement ≥5µm
Nuclear Displacement (µm)
Number of CPs ≥20µm
Proportion of DTCs with any CPs ≥20µm
Mean length (µm) of CPs ≥20µm
Maximum length (µm) of CPs ≥20µm
Age (days)
***
**
*
NS.
n=46
n=43
n=50
n=42
n=37
n=23
n=12
n=14
n=13
n=10

Figure S3

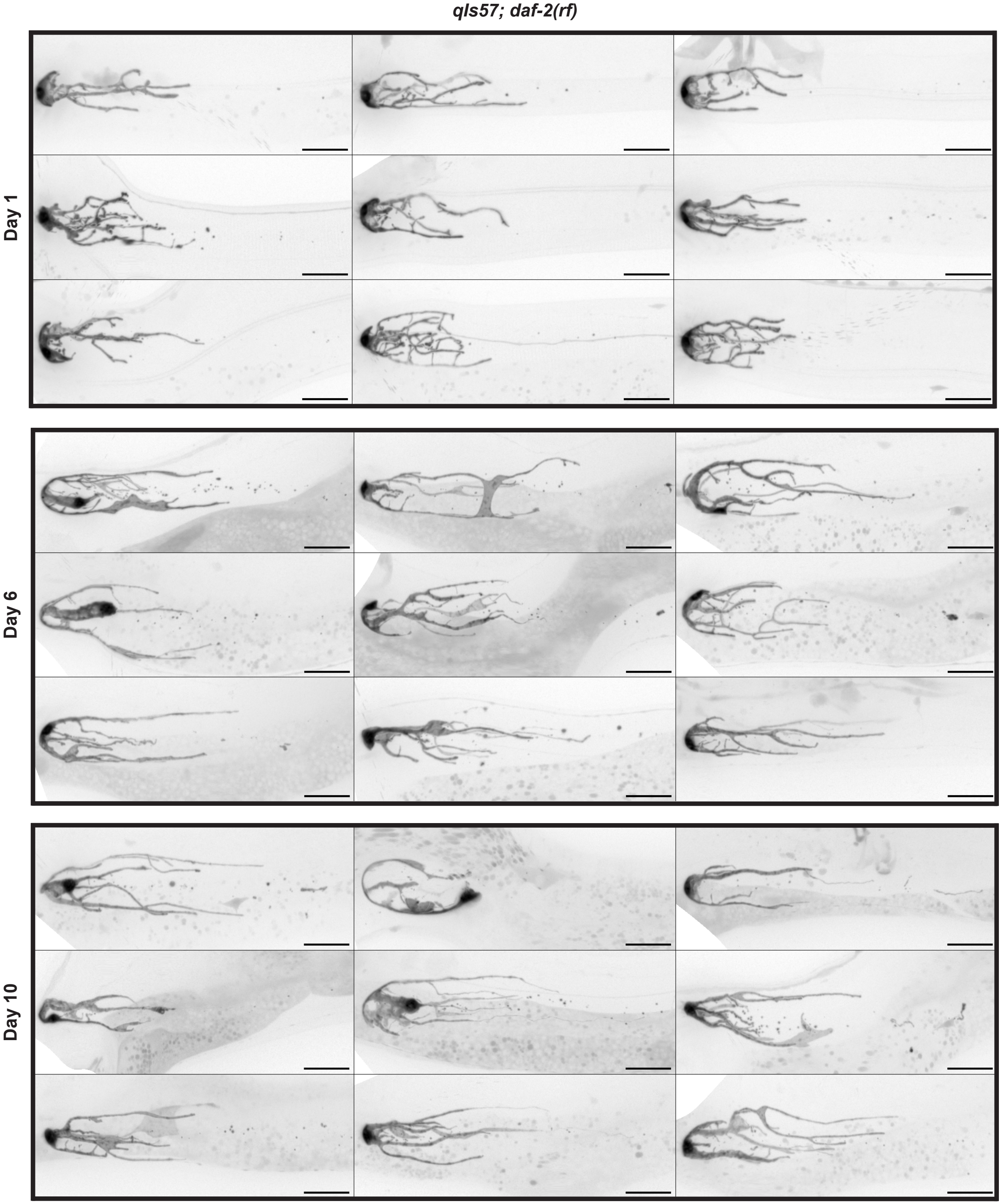

Figure S4

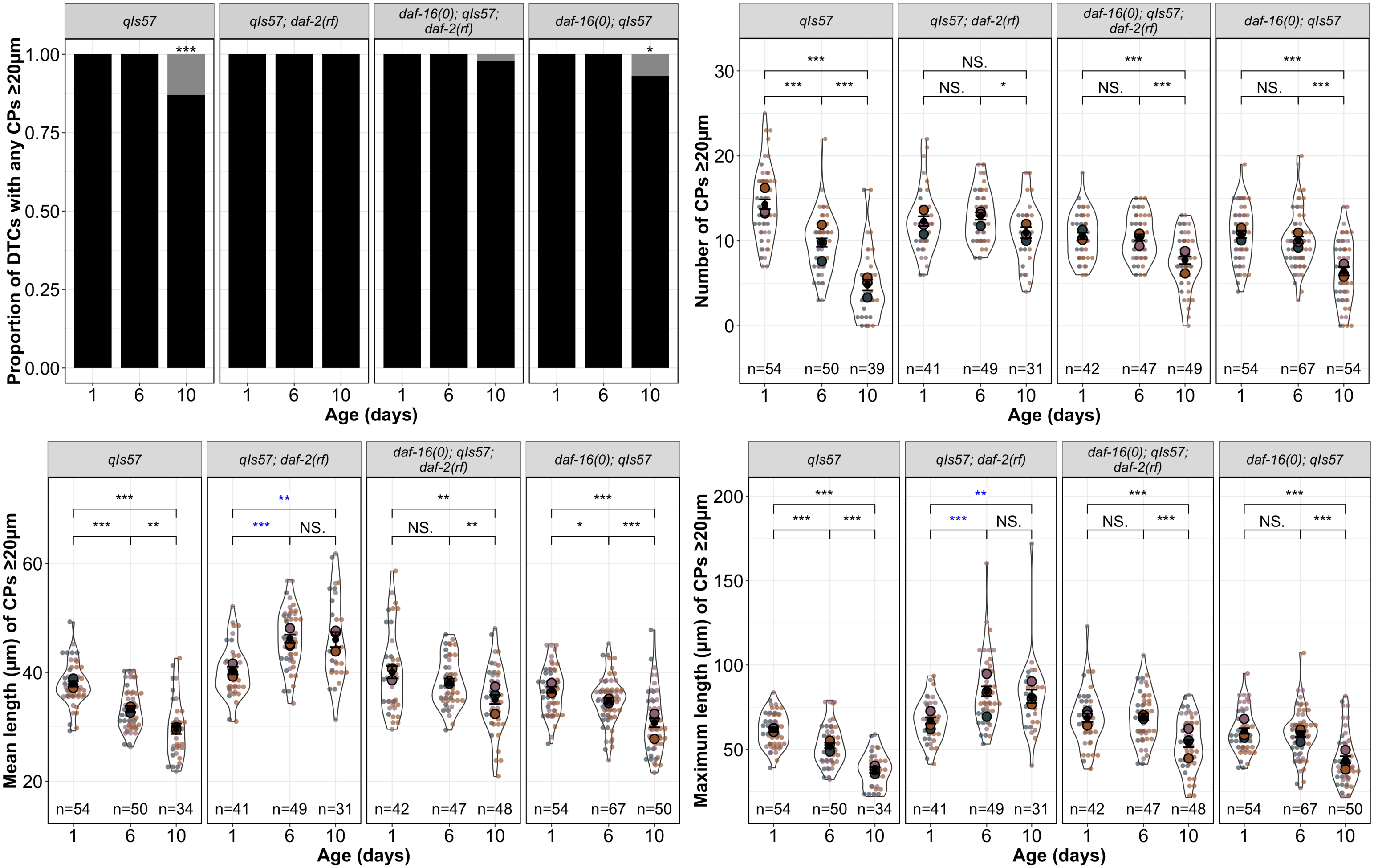
qIs57
qIs57; daf-2(rf)
daf-16(0); qIs57; daf-2(rf)
daf-16(0); qIs57
Proportion of DTCs with any CPs ≥20µm
1.00
0.75
0.50
0.25
0.00
***
*
Age (days)
1
6
10
Number of CPs ≥20µm
30
20
10
0
NS.
n=54 n=50 n=39
n=41 n=49 n=31
n=42 n=47 n=49
n=54 n=67 n=54
Mean length (µm) of CPs ≥20µm
60
40
20
n=54 n=50 n=34
n=41 n=49 n=31
n=42 n=47 n=48
n=54 n=67 n=50
Maximum length (µm) of CPs ≥20µm
200
150
100
50

Figure S5

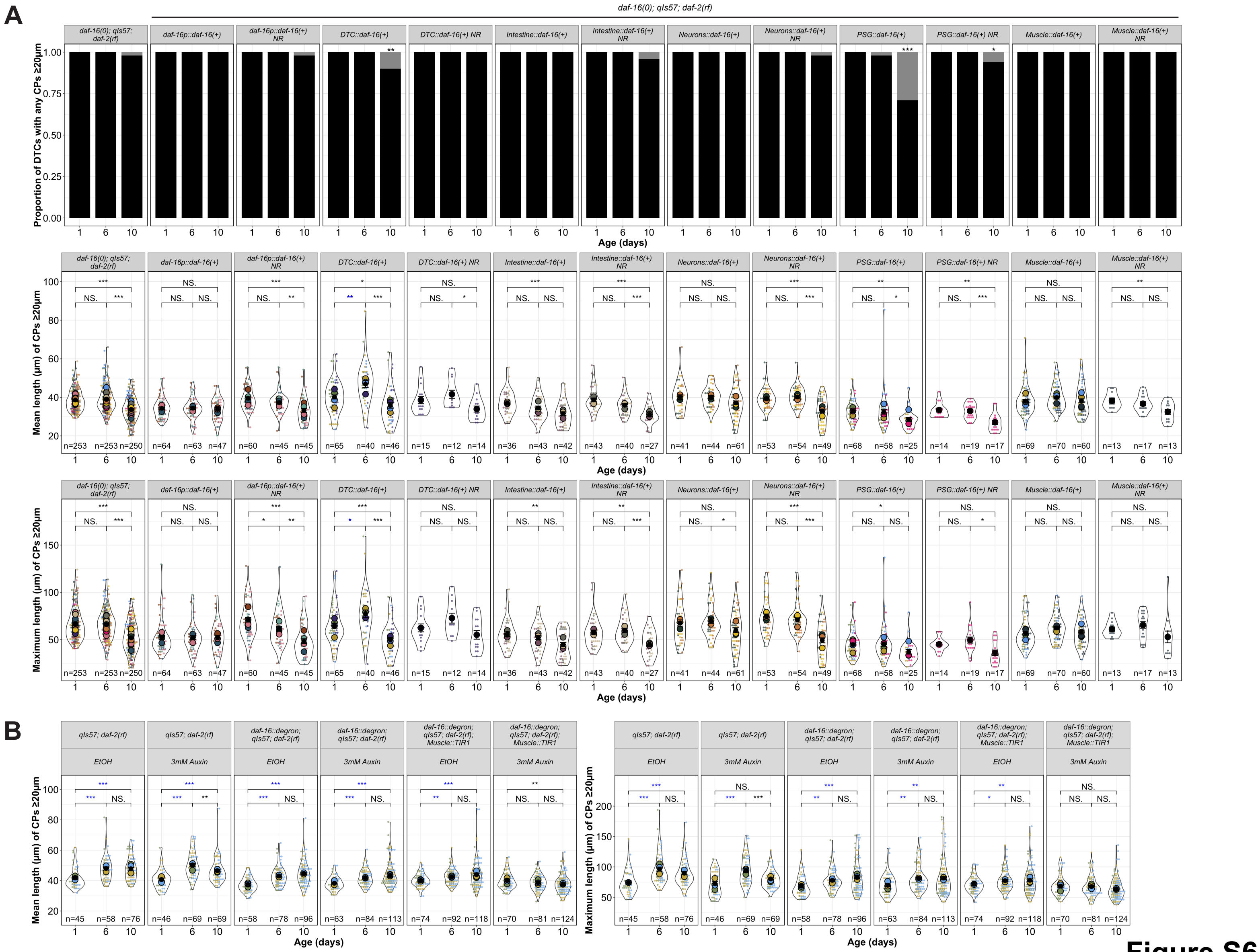
A
daf-16(0); qIs57; daf-2(rf)
daf-16(0); qIs57; daf-2(rf)
daf-16p::daf-16(+)
daf-16p::daf-16(+) NR
DTC::daf-16(+)
DTC::daf-16(+) NR
Intestine::daf-16(+)
Intestine::daf-16(+) NR
Neurons::daf-16(+)
Neurons::daf-16(+) NR
PSG::daf-16(+)
PSG::daf-16(+) NR
Muscle::daf-16(+)
Muscle::daf-16(+) NR
Proportion of DTCs with any CPs ≥20µm
Mean length (µm) of CPs ≥20µm
Maximum length (µm) of CPs ≥20µm
Age (days)
B
qIs57; daf-2(rf)
daf-16::degron; qIs57; daf-2(rf)
daf-16::degron; qIs57; daf-2(rf); Muscle::TIR1
EtOH
3mM Auxin

Figure S6

daf-16(0); qIs57; daf-2(rf); DTC::daf-16(+)

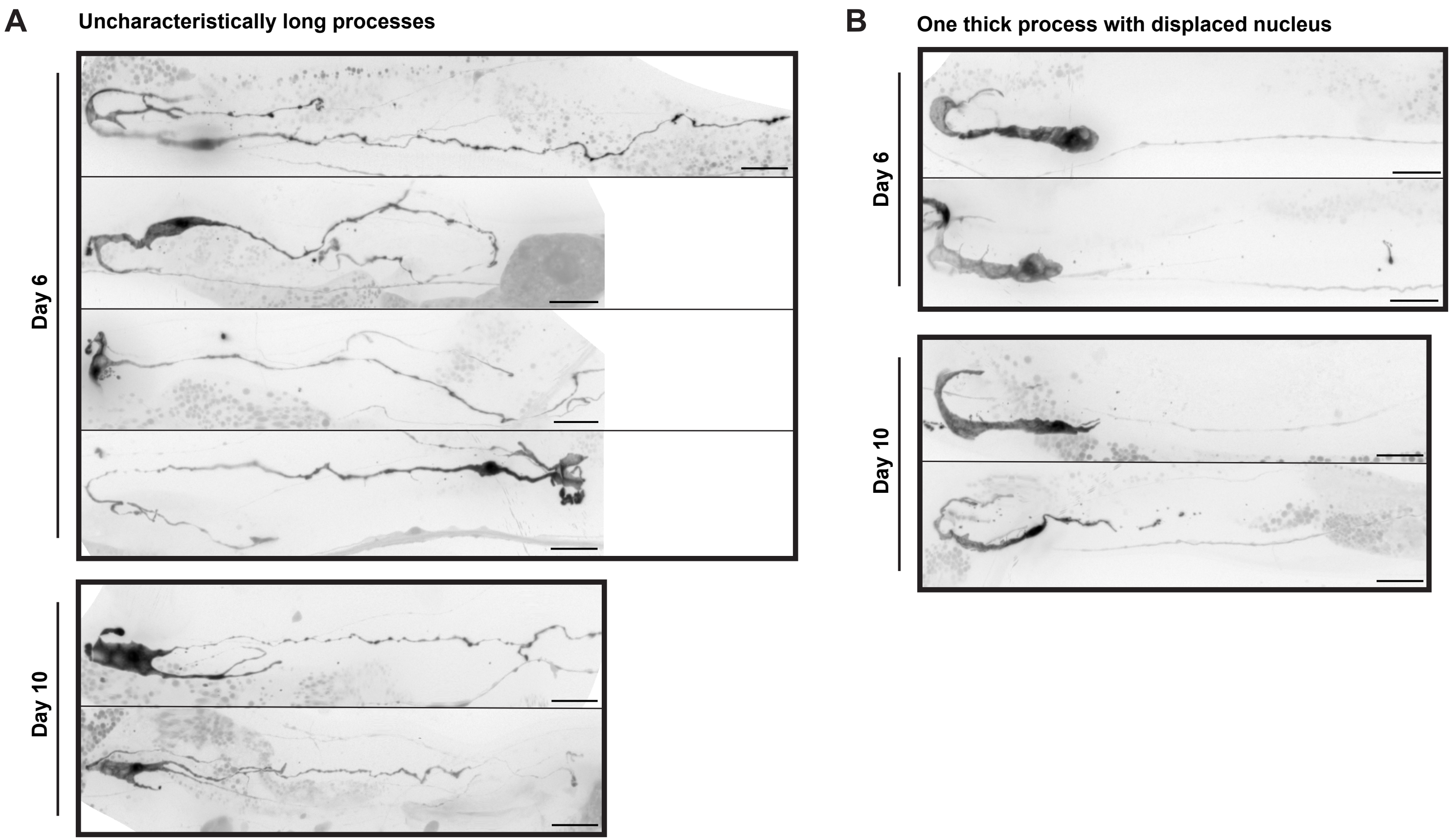

Figure S7

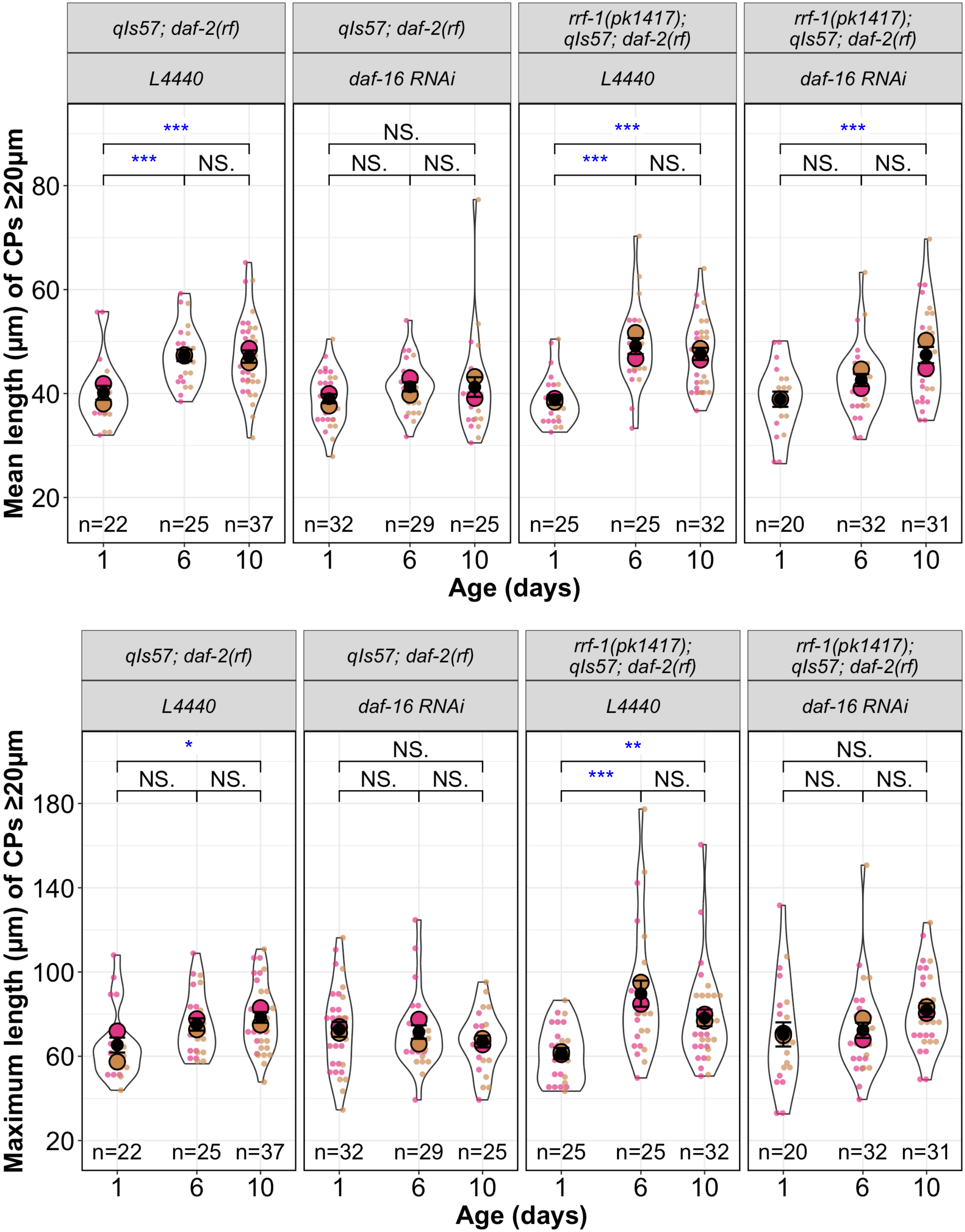
qIs57; daf-2(rf)
L4440
qIs57; daf-2(rf)
daf-16 RNAi
rrf-1(pk1417); qIs57; daf-2(rf)
L4440
rrf-1(pk1417); qIs57; daf-2(rf)
daf-16 RNAi
Mean length (μm) of CPs ≥20μm
***
***
NS.
NS.
NS.
NS.
***
***
NS.
***
NS.
NS.
80
60
40
20
n=22
n=25
n=37
n=32
n=29
n=25
n=25
n=25
n=32
n=20
n=32
n=31
1
6
10
Age (days)
qIs57; daf-2(rf)
L4440
qIs57; daf-2(rf)
daf-16 RNAi
rrf-1(pk1417); qIs57; daf-2(rf)
L4440
rrf-1(pk1417); qIs57; daf-2(rf)
daf-16 RNAi
Maximum length (μm) of CPs ≥20μm
*
NS.
NS.
NS.
NS.
NS.
**
***
NS.
NS.
NS.
NS.
180
140
100
60
20
n=22
n=25
n=37
n=32
n=29
n=25
n=25
n=25
n=32
n=20
n=32
n=31
1
6
10
Age (days)

Figure S8

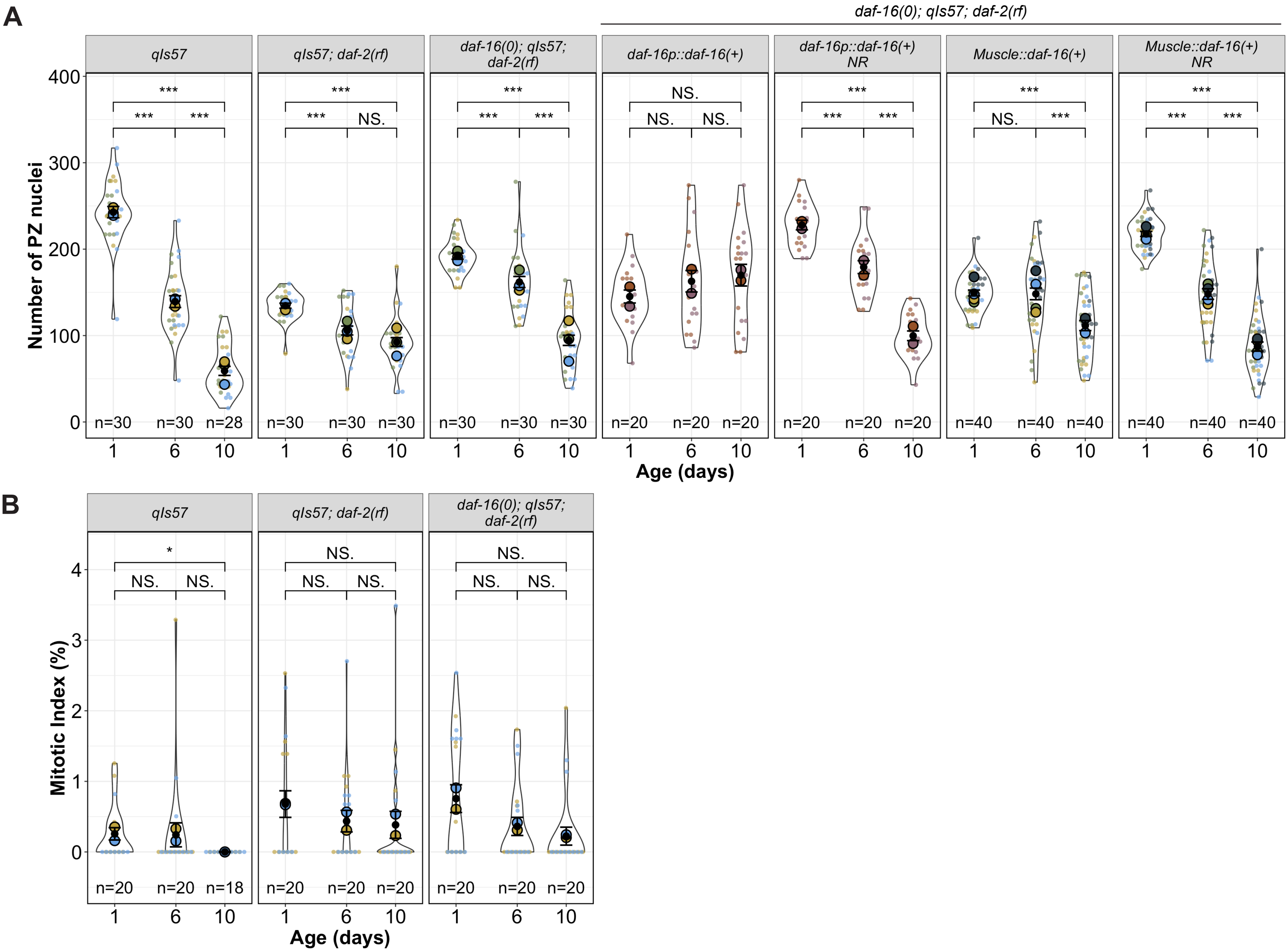
A
daf-16(0); qIs57; daf-2(rf)
qIs57
qIs57; daf-2(rf)
daf-16(0); qIs57; daf-2(rf)
daf-16p::daf-16(+)
daf-16p::daf-16(+) NR
Muscle::daf-16(+)
Muscle::daf-16(+) NR
Number of PZ nuclei
Age (days)
B
Mitotic Index (%)
Age (days)

Figure S9

Supplementary Figure Legends

Figure S1. Comparison of various *lag-2* promoter-driven transgenes
Representative live worm micrographs of DTCs in strains bearing different *lag-2p*-driven fluorescent proteins at Day 1 and Day 10. Relevant genotypes and features for this study are noted. Scale bars are 20 μm.

Figure S2. Examples of DTCs in live worms carrying *qIs57* at Day 1, 6, 10 post mid-L4. Scale bars are 20 μm.

Figure S3. Age-related DTC parameters and effects of *daf-2(rf)* at Days 1, 6, and 10.
(A) DTC features (from top left to bottom right): proportion of DTCs with nuclear displacement ≥5 μm (black bar), position of nuclear displacement (μm) from the distal end, proportion of DTCs with any CPs ≥20 μm (black bar), number of CPs ≥20 μm per DTC, mean and maximum length of CPs ≥20 μm per DTC. N values for proportion plots are the same as for the "Nuclear Displacement" superplot (top right). For superplots, each dot represents a single DTC (n value shown), colors indicate parallel cohorts, large circles in color are averages for each cohort, black dot is pooled average for all cohorts shown, and black line connects pooled average values. N values for mean and maximum length plots reflect only those DTCs with CPs ≥ 20 μm. Day 1 and 10 data are as in Figure 1.
(B) Number, mean and maximum length of CPs per DTC in *daf-2(rf)* at indicated ages in days post mid-L4. Day 1, 10, 20 and 30 data are as in Figure 1. **For all panels**, *qIs57* carries *lag-2p::GFP*, and *daf-2(rf)* is *daf-2(e1370).* See methods for statistics details. For proportion plots, only $p < 0.05$ are indicated; otherwise, NS is "not significant", * $p \geq 0.05$; **$p < 0.01$, *** $p < 0.001$, and blue asterisks indicate a significant increase.

Figure S4. Examples of DTCs in live worms carrying *qIs57* and *daf-2(e1370)* at Day 1, 6, 10 post mid-L4. Scale bars are 20 μm.

Figure S5. Effects of *daf-2* and *daf-16* on DTC process number and length at Days 1, 6, and 10.
DTC features from top to bottom: proportion of DTCs with any CPs beyond 20 μm, number of CPs ≥20 μm per DTC, mean and maximum length of CPs ≥20 μm per DTC at Day 1, 6, and 10 for the indicated genotypes. N values for proportion plots are as in the Number of CPs plot to

the right. For superplots, each dot represents a single DTC n value, colors indicate parallel cohorts, large circles in color are averages for each cohort, black dot is pooled average for all cohorts shown, and black line connects pooled average values. Each cohort average is indicated as well as the pooled average (black). Day 1 and 10 data are as in Figure 2. **For all panels**, *qIs57* carries *lag-2p::GFP*, *daf-16(0)* is *daf-16(mu86),* and *daf-2(rf)* is *daf-2(e1370).* See methods for statistics details; NS is “not significant”, * p < 0.05, ** p < 0.01, *** p < 0.001, and blue asterisks indicate a significant increase.

Figure S6. Effects of tissue restricted *daf-16* on DTC process length with age.
(A) Proportion of DTCs with any CPs ≥20 µm, and mean and maximum length of CPs ≥20 µm for indicated genotypes at Day 1, 6 and 10 post mid-L4 plus additional controls. N values for mean and maximum length plots reflect only those DTCs with CPs ≥ 20 µm. NR indicates “non-Rol” siblings from a Rol (array bearing) mother.
(B) Mean and maximum length of CPs ≥20 µm for conditions indicated where *daf-16::degron* is *hq389[daf-16::GFP::degron]* and *Muscle::TIR1* is *emcSi71[myo-3p::TIR-1::mRuby]*, including EtOH controls. **For all panels,** *qIs57* carries *lag-2p::GFP*, *daf-16(0)* is *daf-16(mu86),* and *daf-2(rf)* is *daf-2(e1370).* For all superplots, colors indicate cohorts within panels; Day 1 and Day 10 data correspond to main Figure 3. See methods for statistics details; NS is “not significant”, * p < 0.05, ** p < 0.01, *** p < 0.001, and blue asterisks indicate a significant increase.

Figure S7. Examples of low-penetrance abnormalities in DTC morphology observed in worms over-expressing *daf-16a* in the DTC. Specific alleles are *daf-16(mu86); qIs57[lag-2p::GFP]; daf-2(e1370); naEx202[lag-2p::daf-16a].* Scale bars are 20 µm.

Figure S8. Effects of *daf-16 RNAi* in *daf-2(e1370)* on Day 1, 6, and 10 on mean and maximum length of CPs. See methods for statistics details; NS is “not significant”, * p < 0.05, ** p < 0.01, *** p < 0.001, and blue asterisks indicate a significant increase. Day 1 and Day 10 data shown are as in main Figure 3B.

Figure S9. Progenitor zone and mitotic index at Day 1, 6, and 10.
Day 1 and Day 10 data correspond to main Figure 4. Additional controls: NR indicates “non-Rol” siblings from a Rol (array bearing) mother. **For all panels,** *qIs57* carries *lag-2p::GFP*, *daf-16(0)* is *daf-16(mu86),* and *daf-2(rf)* is *daf-2(e1370).* For all superplots, colors indicate cohorts within panels. See methods for statistics details; NS is “not significant”, * p < 0.05, *** p < 0.001.

Strains		
***C. elegans* Strains**	**Genotype**	**References**
N2	wild type (Bristol)	Brenner, 1974
CB1370	*daf-2(e1370) III*	Riddle *et al.,* 1981
CF1442	*daf-16(mu86) I; daf-2(e1370) III; muEx169[unc-119p::GFP::daf-16* + pRF4*(rol-6(su1006))]*	Libina *et al.*, 2003
CF1449	*daf-16(mu86) I; daf-2(e1370) III; muEx176[daf-16p::GFP::daf-16* + pRF4*(rol-6(su1006))]*	Libina *et al.,* 2003
CF1514	*daf-16(mu86) I; daf-2(e1370) III; muEx211[*pNL213*(ges-1p::GFP::daf-16)* + pRF4*(rol-6(su1006))]*	Libina *et al.*, 2003
CF1515	*daf-16(mu86) I; daf-2(e1370) III; muEx212[*pNL212*(myo-3p::GFP::daf-16)* + pRF4*(rol-6(su1006))]*	Libina *et al.,* 2003
GC832	*glp-1(e2141) III*	Priess *et al.*, 1987; Hutter and Schnabel, 1994;<br>Dalfó *et al*., 2010
GC1019	*rrf-1(pk1417) I; daf-2(e1370) III*	Michaelson *et al.,* 2010
GC1109	*daf-16(m26) I; daf-2(e1370) III; naEx202[*pGC461*(lag-2p::daf-16::GFP)* + pRF4*(rol-6(su1006))]*	Michaelson *et al.,* 2010
GC1172	*xnSi1[mex-5p::GFP::PHdomain::nos-2 3'UTR] II ; naIs37[*pGC457*(lag-2p::PHdomain::mCherry-unc-119(+))]*	Chihara & Nance, 2012;<br>Pekar *et al.,* 2017
GC1285	*daf-16(m26) I; daf-2(e1370) III; naEx239[*pGC629*(fos-1p::GFP::daf-16)* + pRF4*(rol-6(su1006))]*	Qin & Hubbard, 2015
GC1332	*daf-16(mu86) I; daf-2(e1370) III*	Qin & Hubbard, 2015
GC1412	*naSi8[*pGC680*(lag-2p(3kb)::GFP-PH::let-858(3'))] II*	Pekar *et al.,* 2017
GC1593	*qIs57[pJK590(lag-2p::GFP)] II; glp-1(e2141) III*<br><br>N2 males were crossed with JK2869 hermaphrodites, and the resulting heterozygous males were crossed with GC832 hermaphrodites. *qIs57* was followed visually. g*lp-1(e2141)* was confirmed by DNA sequence analysis.	This study
GC1607	*qIs57[*pJK590*(lag-2p::GFP)] II; daf-2(e1370) III*<br><br>JK2869 males were crossed with CB1370 hermaphrodites, and *qIs57* was followed visually. *daf-2(e1370)* was followed by the Daf-c phenotype and subsequent genotyping by DNA sequence analysis.	This study
GC1616	*daf-16(mu86) I; qIs57[*pJK590*(lag-2p::GFP)] II; daf-2(e1370) III*<br><br>GC1332 males were crossed with GC1607 hermaphrodites, and *qIs57* was followed visually. The presence of both *daf-16(mu86)* and *daf-2(e1370)* were followed by monitoring the Daf-c phenotype and subsequent DNA sequence analysis.	This study
GC1676	*daf-16(mu86) I; qIs57[*pJK590*(lag-2p::GFP)] II; daf-2(e1370) III; naEx202[*pGC461*(lag-2p::daf-16::GFP)* + pRF4*(rol-6(su1006))]*<br><br>GC1616 males were crossed with GC1109 array-bearing (Rol) hermaphrodites, and *qIs57* was followed visually. *daf-16(mu86)* was followed by DNA sequence analysis. DTC-specific expression of *daf-16a(+)* was confirmed visually by presence of GFP in DTC nuclei and some neuronal nuclei.	This study
GC1682	*daf-16(mu86) I; qIs57[*pJK590*(lag-2p::GFP)] II; daf-2(e1370) III; muEx212[*pNL212*(myo-3p::GFP::daf-16)* + pRF4*(rol-6(su1006))]*<br><br>GC1616 males were crossed with CF1515 array-bearing (Rol) hermaphrodites, and *qIs57* was followed visually. *daf-16(mu86)* was followed by DNA sequence analysis. Muscle-specific expression of daf-16a(+) was confirmed by presence of GFP in muscle nuclei.	This study

GC1683	*daf-16(mu86) I; qIs57[*pJK590*(lag-2p::GFP)] II; daf-2(e1370) III; naEx239[*pGC629*(fos-1p::GFP::daf-16)* + pRF4*(rol-6(su1006))]*<br><br>GC1616 males were crossed with GC1285 array-bearing (Rol) hermaphrodites, and *qIs57* was followed visually. *daf-16(mu86)* was followed by DNA sequence analysis. PSG-specific expression of *daf-16a(+)* confirmed visuallly by presence of GFP in PSG nuclei.	This study
GC1691	*daf-16(mu86) I; qIs57[pJK590(lag-2p::GFP)] II; daf-2(e1370) III; muEx176[daf-16p::GFP::daf-16a* + pRF4*(rol-6(su1006))]*<br><br>GC1616 males were crossed with CF1449 array-bearing (Rol) hermaphrodites, and *qIs57* was followed visually. *daf-16(mu86)* was followed by DNA sequence analysis. Expression of *daf-16a(+)* was confirmed visually by the presence of GFP in multiple tissues.	This study
GC1692	*daf-16(mu86) I; qIs57[*pJK590*(lag-2p::GFP)] II; daf-2(e1370) III; muEx211[*pNL213*(ges-1p::GFP::daf-16)* + pRF4*(rol-6(su1006))]*<br><br>GC1616 males were crossed with CF1514 array-bearing (Rol) hermaphrodites, and *qIs57* was followed visually. *daf-16(mu86)* was followed by DNA sequence analysis. Intestine-specific expression of *daf-16a(+)* was confirmed visually by presence of GFP in intestinal nuclei.	This study
GC1696	*daf-16(mu86) I; qIs57[*pJK590*(lag-2p::GFP)] II; daf-2(e1370) III; muEx169[unc-119p::GFP::daf-16* + pRF4*(rol-6(su1006))]*<br><br>GC1616 males were crossed to CF1442 array-bearing (Rol) hermaphrodites, and *qIs57* was followed visually. *daf-16(mu86)* was followed by DNA sequence analysis. Neuron-specific expression of *daf-16a(+)* was confirmed visually by presence of GFP in neuronal nuclei.	This study
GC1778	*daf-16(mu86) I; qIs57*[pJK590*(lag-2p::GFP)*] *II*<br><br>JK2869 males were crossed with GC1616 hermaphrodites. *daf-2(e1370)* was selected against by absence of the Daf-c phenotype. Both *daf-2(e1370)* and *daf-16(mu86)* were subsequently confirmed by DNA sequence analysis.	This study
GC1817	*daf-16(hq389[daf-16::GFP::degron]) I; qIs57[*pJK590*(lag-2p::GFP)] II; daf-2(e1370) III; emcSi71[myo-3p::TIR-1::mRuby] (IV: -0.05)*<br><br>HAL230 males were crossed with GC1819 hermaphrodites, and *qIs57* was followed visually. *daf-2(e1370)* was followed by the Daf-c phenotype and subsequent genotyping by DNA sequence analysis. *daf-16::GFP::degron* was confirmed visually by the presence of GFP in the nuclei of multiple tissues and by subsequent genotyping by DNA sequence analysis. *myo-3p::TIR1::mRuby* was confirmed visually by the presence of red fluoresence in muscle nuclei and by subsequent genotyping by DNA sequence analysis.	This study
GC1819	*daf-16(hq389[daf-16::GFP::degron]) I; qIs57[*pJK590*(lag-2p::GFP)] II; daf-2(e1370) III*<br><br>GC1607 males were crossed with MQD2499 hermaphrodites, and *qIs57* was followed visually. *daf-2(e1370)* was followed by the Daf-c phenotype. *daf-16::GFP::degron* was confirmed visually by the presence of GFP in the nuclei of multiple tissues. *myo-3p::TIR1::mRuby* was selected against by the absence of red fluorescence in the muscle. All three alleles were confirmed by subsequent genotyping by DNA sequence analysis.	This study
GC1912	*rrf-1(pk1417) I; qIs57[*pJK590*(lag-2p::GFP)] II; daf-2(e1370) III*<br><br>GC1607 males were crossed with GC1019 hermaphrodites, and *qIs57* was followed visually. *daf-2(e1370)* was followed by the Daf-c phenotype and by subsequent genotyping by DNA sequence analysis. *rrf-1(pk1417)* was also confirmed by DNA sequence analysis.	This study
HAL230	*unc-119(ed3) III; emcSi71[myo-3p::TIR1::mRuby] (IV: -0.05)*	Sabatella *et al.,* 2021
JK2869	*qIs57[*pJK590*(lag-2p::GFP)] II*	Siegfried *et al.,* 2004
JK4472	*qIs154[lag-2p::MYR::tdTomato + ttx-3p::GFP] V*	Byrd *et al.,* 2014

MQD2499	*daf-16(hq389[daf-16::GFP::degron]) I; hqSi10[myo-3p::TIR1::mRuby::unc-54 3' UTR + Cbr-unc-119(+)] II; daf-2(e1370) unc-119(ed3) III*	Zhang *et al.*, 2022
PD8488	*rrf-1(pk1417) I*	Miller *et al.*, 2003
**Bacterial Strains**	**Genotype**	**References**
E. coli HT115(DE3)	[F-, mcrA, mcrB, IN(rrnD-rrnE)1, rnc14::Tn10(DE3 lysogen: lacUV5 promoter -T7 polymerase]	Timmons *et al.*, 2001
E. coli HT115(DE3) L4440	HT115(DE3) bacteria bearing the L4440 "empty vector" plasmid	Timmons *et al.*, 2001
E. coli HT115(DE3) 10020-B-8	HT115(DE3) bearing a plasmid that produces dsRNA corresponding to the *daf-16* cDNA	Rual *et al.*, 2004
E. coli OP50	*E. coli* Uracil auxotroph	CGC

Oligonucleotides

Name	Sequence	Gene
GCo969	GCCGCACAGATTTGTGATGGTATGGCG	F primer for *daf-2(e1370)*
GCo970	TCATCAAGATCCAGTGCTTCTGAATCG	R primer for *daf-2(e1370)*
GCo1217	CTCGCAAATGGATATCCCGG	F primer for *glp-1(e2141)*
GCo1354	CATCCTTCCTTAGCGGCAAGC	R primer for *glp-1(e2141)*
GCo1624	CGAATCAGTTTTTTTTCTTCACTCGCC	F primer for *daf-16(mu86)*
GCo1625	CGTGAGAAATCGTTTGAATCGATCCGGC	R primer for *daf-16(mu86)*
GCo1971	TAGCTATCAAATCCAATACTGGGAG	F primer for *rrf-1(pk1417)*
GCo1976	GTTGTGGGAATACTACTACATCAC	R primer for *rrf-1(pk1417)*
GCo2949	CAGCCTATACGGGAGCAATGAGCA	F primer for *daf-16(m26)*
GCo2950	GCGTTCCTATACGAATGAAACCAACTGG	R primer for *daf-16(m26)*
GCo3177	CCTCTACTTCTGCCGTCAAATGACCAACG	F primer for *TIR1*
GCo3178	ACAGATCCCTCAAGAACAACCTTCATGCG	R primer for *TIR1*
GCo3183	TTCAGAATGCCACTACCGGGTGCCTAT	F primer for *daf-16::degron*
GCo3184	TGTTTGCAGTTCTAAGGCCCTTATTGGA	R primer for *daf-16::degron*
GCo3225	TGGCTCATTCCTTTATCGTCATACA	F primer for *rde-1(ne300)*
GCo3226	CGAGTATCAATCCAGGTGGAACTAT	R primer for *rde-1(ne300)*

Reagents

Name	Source	Identifier
Indole-3-acetic acid (Auxin)	Thermo Scientific	A10556.14
VECTASHIELD® Antifade Mounting Medium, With DAPI, Liquid	Vector Laboratories	H-1200-10
β-Lactose	Sigma-Aldrich	L3750

Supplementary references

Brenner, S. (1974). The genetics of Caenorhabditis elegans. *Genetics* **77**, 71-94.

Byrd, D. T., Knobel, K., Affeldt, K., Crittenden, S. L. and Kimble, J. (2014). A DTC niche plexus surrounds the germline stem cell pool in Caenorhabditis elegans. *PLoS One* **9**, e88372.

Chihara, D. and Nance, J. (2012). An E-cadherin-mediated hitchhiking mechanism for C. elegans germ cell internalization during gastrulation. *Development*.

Dalfo, D., Priess, J. R., Schnabel, R. and Hubbard, E. J. A. (2010). glp-1(e2141) sequence correction. In *Worm Breeder's Gazette*.

Hutter, H. and Schnabel, R. (1994). glp-1 and inductions establishing embryonic axes in C. elegans. *Development* **120**, 2051-2064.

Libina, N., Berman, J. R. and Kenyon, C. (2003). Tissue-specific activities of C. elegans DAF-16 in the regulation of lifespan. *Cell* **115**, 489-502.

Michaelson, D., Korta, D. Z., Capua, Y. and Hubbard, E. J. A. (2010). Insulin signaling promotes germline proliferation in C. elegans. *Development (Cambridge, England)* **137**, 671-680.

Miller, M. A., Ruest, P. J., Kosinski, M., Hanks, S. K. and Greenstein, D. (2003). An Eph receptor sperm-sensing control mechanism for oocyte meiotic maturation in Caenorhabditis elegans. *Genes & development* **17**, 187-200.

Pekar, O., Ow, M. C., Hui, K. Y., Noyes, M. B., Hall, S. E. and Hubbard, E. J. A. (2017). Linking the environment, DAF-7/TGFbeta signaling and LAG-2/DSL ligand expression in the germline stem cell niche. *Development* **144**, 2896-2906.

Priess, J. R., Schnabel, H. and Schnabel, R. (1987). The glp-1 locus and cellular interactions in early C. elegans embryos. *Cell* **51**, 601-611.

Qin, Z. and Hubbard, E. J. A. (2015). Non-autonomous DAF-16/FOXO activity antagonizes age-related loss of C. elegans germline stem/progenitor cells. *Nature Communications* **6**, 7107.

Riddle, D. L., Swanson, M. M. and Albert, P. S. (1981). Interacting genes in nematode dauer larva formation. *Nature* **290**, 668-671.

Rual, J. F., Ceron, J., Koreth, J., Hao, T., Nicot, A. S., Hirozane-Kishikawa, T., Vandenhaute, J., Orkin, S. H., Hill, D. E., van den Heuvel, S., et al. (2004). Toward improving Caenorhabditis elegans phenome mapping with an ORFeome-based RNAi library. *Genome Res* **14**, 2162-2168.

Sabatella, M., Thijssen, K. L., Davo-Martinez, C., Vermeulen, W. and Lans, H. (2021). Tissue-Specific DNA Repair Activity of ERCC-1/XPF-1. *Cell Rep* **34**, 108608.

Siegfried, K. R., Kidd, A. R., 3rd, Chesney, M. A. and Kimble, J. (2004). The sys-1 and sys-3 genes cooperate with Wnt signaling to establish the proximal-distal axis of the Caenorhabditis elegans gonad. *Genetics* **166**, 171-186.

Timmons, L., Court, D. L. and Fire, A. (2001). Ingestion of bacterially expressed dsRNAs can produce specific and potent genetic interference in Caenorhabditis elegans. *Gene* **263**, 103-112.

Zhang, Y. P., Zhang, W. H., Zhang, P., Li, Q., Sun, Y., Wang, J. W., Zhang, S. O., Cai, T., Zhan, C. and Dong, M. Q. (2022). Intestine-specific removal of DAF-2 nearly doubles lifespan in Caenorhabditis elegans with little fitness cost. *Nat Commun* **13**, 6339.
